# Fields and Forests in Flames: Lead and Mercury Emissions from Wildfire Pyrogenic Activity

**DOI:** 10.1289/ehp.1104672

**Published:** 2012-02-01

**Authors:** Louise J. Kristensen, Mark P. Taylor

**Affiliations:** Environmental Science, Macquarie University, Sydney, Australia, E-mail: mark.taylor@mq.edu.au

In the article “Fields and Forests in Flames,” Weinhold (2011) addressed the toxic health effects associated with fire smoke. Although he acknowledged the limited data on the toxicity of wildfires, several important studies on environmental emissions from fire events and their consequences were omitted.

Weinhold (2011) listed multiple compounds from wildfires, back burning, and incinerated buildings, but listed only four elements: potassium, chlorine, sulfur, and silicon. Significant omissions were the toxic elements lead and mercury. Lead has been identified as one of the most environmentally pervasive and damaging metals to human health (Patterson 1965).

Several studies have detailed the remobilization of metals from fire events (e.g., Finley et al. 2009; Nriagu 1989; Odigie and Flegal 2011; Young and Jan 1977). These studies showed that significant levels of toxic and nontoxic metals are emitted into the environment during fires. Young and Jan (1977) found that smoke from a 1975 Californian wildfire emitted various metals, including cadmium, chromium, copper, iron, lead, manganese, nickel, silver, and zinc, up to 100 km from the fire. Contamination of local marine waters with lead, iron, and manganese from the wildfire exceeded the polluting effects of the local municipal wastewater, the main source of metals.

Nriagu (1989) and Finley et al. (2009) estimated that the amount of lead (plus other trace metals) and mercury, respectively, from fires were comparable to emissions from anthropogenic sources such as industrial processes and city pollution. Nriagu (1989) estimated that global emissions of lead from wildfires ranged from 60,000 to 3,800,000 kg/year, with an average of 1,900,000 kg/year. Global mercury emissions from wildfires are also significant, estimated at 890,000 ± 490 kg/year for gaseous elemental mercury and 170 ± 100 kg/year for particulate-bound mercury (Finley et al. 2009). Until recently it was not known whether lead released by wildfires is from natural and or industrial sources. Odigie and Flegal (2011) measured the isotopic lead composition of ash from the 2009 Jesusita Fire in Southern California. Their work showed clearly that the ash from the wildfire contained industrial lead primarily from leaded gasoline used in Southern California during the 1960s through the 1980s.

Environmental media, such as air, dust, sediment, soil, and water, have well-defined and strict environmental and human health guidelines because of their damaging effect on natural and anthropogenic systems. Even low levels of atmospheric lead emissions are known to cause adverse human health effects, including irreversible neurological damage. For example, the U.S. Environmental Protection Agency (EPA) recently reduced the lead-in-air guideline by an order magnitude—from 1.5 µg/m^3^ to 0.15 µg/m^3^—after reviewing > 6.000 human health–lead-related studies (U.S. EPA 2008). Although pyrogenic activity affects environmental quality, its effects remain ill-defined, despite evidence of harmful human health effects from exposure to toxicants, even at very low levels (Lanphear et al. 2005). The risk from fires is likely to increase as the frequency of climatically driven fire events rises in response to predicted global warming (Intergovernmental Panel on Climate Change 2007). The predicted environmental changes present a significant research opportunity for those interested in monitoring the biogeochemical cycling of metals and their potential risk of harm to human and environmental health systems.
